# Male Mating Expectations in Brazilian and American Samples

**DOI:** 10.3389/fpsyg.2021.617754

**Published:** 2021-02-10

**Authors:** Felipe Nalon Castro, Wallisen Tadashi Hattori, Steven J. C. Gaulin, Maria Emília Yamamoto, Fívia de Araújo Lopes

**Affiliations:** ^1^Human Behavior Evolution Laboratory, Psychobiology Postgraduate Program, Department of Physiology and Behavior, Federal University of Rio Grande do Norte, Natal, Brazil; ^2^Department of Public Health, Faculty of Medicine, Federal University of Uberlândia, Uberlândia, Brazil; ^3^Department of Anthropology, University of California, Santa Barbara, Santa Barbara, CA, United States

**Keywords:** cross-cultural, mate value, reproduction, mating preference, men

## Abstract

This study aims to investigate assortative mating based on mate value from male perspective. Male participants (132 Brazilian and 106 American) evaluated hypothetical “stimulus” males described in terms of physical attractiveness, social skills, and social status (each varied in high or low levels). Participants rated each stimulus and each stimulus' preferred mating partner on nine traits. The results showed that (1) positive assortative mating was expected in romantic relationships; (2) the stimulus ratings did not vary independently, suggesting that mate value is the result of the interaction of the characteristics of individuals; and (3) that participants expected physically attractive and healthier female partners to pair with high-status male stimuli. The American and Brazilian mating expectations were similar, minor differences indicate that Brazilian participants considered men with high levels of social skills to be more ambitious and intelligent; American participants expected men of high status to be healthier; Brazilians expect men of high status to have more attractive faces, while Americans expected these men to possess more attractive bodies; and Brazilian participants assigned more attractive bodies to men of lower status. These differences reflect the influence of economic and cultural factors on the local environment. The study contributes to the understanding of the construction of market value and reveals that male expectations are in line with human mating preferences. The investigation of mating expectations can be a rich approach to investigate socio-cultural aspects that are related to mating in different cultures

## Introduction

Usually, males and females invest differently in their young and the asymmetry in parental investment impacts reproductive behavior. As a result, higher levels of intrasexual competition in males and intersexual selection in females are observed, affecting the traits that constitutes mate value (Trivers, 1972; Buss and Schmitt, 2019). Mate value can be defined as the set traits an individual possesses that impacts their reproductive outcome (traits that affects finding, attracting, and retaining a mate) (Figueredo et al., 2006; Fisher et al., 2008).

Among human, women mating value is related to reproductive potential and maternal care (Chang et al., 2017). From an evolutionary perspective, male preferences have been designed to track female reproductive value (*sensu* Fisher, 1930), a measure of future reproductive potential, rather than current health or fertility (Lassek and Gaulin, 2018a,b). Signs of nubility, such as a youthful appearance, seem to be most important in shaping men's preferences (Symons, 1979, 1995; Fessler et al., 2005; Sugiyama, 2005; Andrews et al., 2017; Lassek and Gaulin, 2019).

Male mating value is associated to men's physical fitness, health, and ability to provide. In addition, men have paternal care, so parenting skills and pair bonding have become relevant in the evaluation of male attributes (Lu et al., 2015; Chang et al., 2017). Because woman invest a great deal in their children, they might usefully discriminate among potential partners based on time and energy resources men can provide to their partners and children (Buss and Schmitt, 1993; Pawlowski, 2000; Geary et al., 2004; Wang et al., 2018). Thus, characteristics that signify social compatibility, commitment and positive disposition toward parental investment are most highly valued by women (Geary et al., 2004; Brase, 2006; Castro et al., 2012).

According to Noë and Hammerstein (1995), biological-market theory applies to situations where there are at least two classes of “traders” exchanging commodities in a way that is mutually beneficial, in which individuals outbid their competitors to acquire the highest quality partner, and trades can only be completed via consent. In romantic relationships, men and women seek social alliance and reproductive opportunities, compete within their own gender-class over access to high-quality partners, and display their more attractive characteristics. Couples emerge from the process of mutually evaluating the characteristics of potential partners (Pawlowski, 2000; Conroy-Beam et al., 2019).

Mate preferences can be classified into relative and absolute (Figueredo et al., 2006). Relative preferences are those that an individual presents and has their own characteristics as a reference. Mate choice can result in pairs of individuals who have similar or different characteristics (respectively positive and negative assortative mating), as long as they have a similar mate value (Luo, 2017). Mate preferences are also expected to adjust to the local environment and achieve better reproductive outcomes (Gangestad and Simpson, 2000; Pillsworth, 2008; Noë, 2017; Luberti et al., 2020). Adjustment to ecological and cultural factors could generate consensus on preferences and absolute preferences, those preferences observed to be similar across all individuals (Figueredo et al., 2006), could emerge. Social consensus on how couples should be formed, and typical sexual preferences, can in turn originate mate expectations, that is, ideal standards about the traits that men and women should present for establishing romantic relationships.

The current study aims to investigate assortative mating based on mate value from male perspective. We hypothesized that similarity should be expected in couples. Men who have a high level of a given characteristic (e.g., social skills) should have partners with high scores for those same characteristic. We assume that the possession of a given characteristic will increase the evaluation of different characteristics in men and their partners. This hypothesis generates two predictions: (1) men who have high level of a given characteristic (e.g., physical attractiveness) will be assigned a high rating on other traits (e.g., traits related to social skills); (2) there is an expectation that men who have characteristics most valued by women for long-term relationships (e.g., resources and social skills) would be paired with women who have traits most valued by men (e.g., attractive face and body). Finally, we expected that male expectations will be the same for the American and Brazilian sample because they might derive from evolutionary universal adaptations.

## Methods

### Participants

The study had the participation of 238 male undergraduate students. Detailed information of the two samples can be seen in Table 1.

**Table 1:** Sample characteristics.

	**Brazilian sample**	**American sample**
Number of participants	132	106
Age (years)	*M* = 21.89	*M* = 18.84
	*SD* = 2.68	*SD* = 1.56
Skin color^*^	50.8% white	35.8% European
	35.6% pardo^**^	18.9% Asian
	6.8% yellow^***^	18.0% Hispanic
	4.5% black	17.0% mixed ethnicity
	2.3% indigenous	2.8% African American
		7.5% Other category
Sexual orientation	84.0% attracted to the opposite sex	88.6% attracted to the opposite sex
	9.9% attracted to the same sex	7.6% attracted to both the sexes
	6.1% attracted to both sexes	3.8% attracted to the same sex
Participant institution	Universidade Federal do Rio Grande do Norte, Natal (northeastern Brazil)	University of California, Santa Barbara (United States)

* Classified according of skin color classification used in Brazil [Instituto Brasileiro de Geografia e Estatística (IBGE), 2010];

** mixed-race individuals;

*** *Asian descent: Japanese, Chinese, and Korean*.

### Procedure

Participants responded in person to an anonymous individual questionnaire that presented descriptions of eight different hypothetical men; these men were the “stimulus subjects” (SS). Participants were asked to: (1) rate each hypothetical male, and (2) rate how they imagined the corresponding probable partner (PP) (hypothetical women) of each SS, on the same set of nine traits (details below). The participants were then asked to provide some demographic information. The text preceding the descriptions was as follows: *In the following pages you will be introduced to eight people. Please read the description of each person. Afterward, you will be asked to describe the person; next you will be asked to describe the person's probable partner*. The individual questionnaires were applied collectively in the classroom or in the laboratory. Participants were unable to interact with their peers and the experimenter was on site.

### Stimulus Subject Descriptions

The eight SS descriptions were presented in paragraph format and were developed to include all possible combinations of high vs. low values of three characteristics: physical attractiveness, social skills and current/prospective social status, yielding a 2 × 2 × 2 within-subjects design (Table 2; Supplementary Table 1). The subject descriptions were presented in random order to each of the participants, who used a 10-point Likert scale to rate each of the eight SS on nine separate traits: attractive face, attractive body, good health, sociability, agreeableness, sincerity, good financial status, ambitious/hard working, and intelligence. These traits describe relevant dimensions of the mating value for humans (Castro and Lopes, 2011; Lu et al., 2015; Chang et al., 2017; Buss and Schmitt, 2019; Thomas et al., 2019). Participants also predicted a likely PP for each SS, using the same scales and traits. This study design replicates the methodology described by Castro et al. (2018) in their investigation of the female mating expectations.

**Table 2:** Stimulus descriptions by characteristic levels.

**Stimulus**	**Physical attractiveness**	**Social skills**	**Social status**
SS1	High	High	High
SS2	High	High	Low
SS3	High	Low	High
SS4	High	Low	Low
SS5	Low	High	High
SS6	Low	High	Low
SS7	Low	Low	High
SS8	Low	Low	Low

*The description code refers to the code used to rate the traits of the stimulus subject (SS)*.

### Analyses

As our participants acted as “third parties” in a task of imaginary couple formation, all sexual-orientation groups were included in the data analysis. To investigate the effects of high/low physical attractiveness, social skills, and social status on the SS and PP ratings, the overall mean value for each trait was used to evaluate SS and PP separately and calculated for: the descriptions formulated with high (SS1, SS2, SS3, and SS4) and low levels of physical attractiveness (SS1, SS2, SS3, and SS4); high (SS1, SS2, SS5, and SS6) and low levels of social skills (SS3, SS4, SS7, and SS8); and high (SS1, SS3, SS5, and SS7) and low levels of social status (SS2, SS4, SS6, and SS8). For the Brazilian and USA samples, we used mixed General Linear Models [GLM] to assess the effects of high vs. low levels (of physical attractiveness, social skills, and social status) on mean Likert ratings of each of the nine traits for both SS and PP. In the GLM tests, the ratings of each trait were treated as dependent variables, while profiles (high vs. low) and samples (Brazilian vs. American) were fixed factors. Separate analyses were performed to contrast the different levels of physical attractiveness, social skills and social status. Since each SS and PP were rated on the combination of nine traits, the significance level was set at 0.0056 (0.05 ÷ 9) for the analysis of each trait (avoiding type 1 errors). In all analyses, the Pearson's correlation coefficient was calculated as a measure of effect size to allow comparisons of the magnitude of the significant effects found. In this study, effects sizes >0.70 were considered large effects, effects sizes ranging from 0.40 to 0.69 were medium effects, and effect sizes lower than 0.39 small effects.

## Results

### Brazilian and American Samples Considered Together

**Figure 1:**
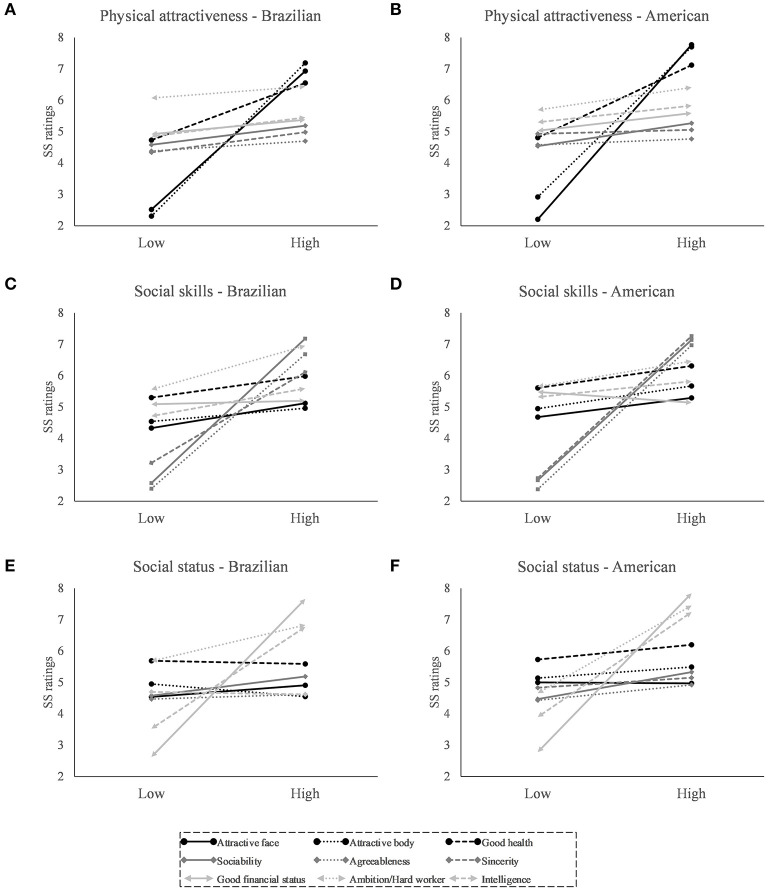
Means of traits of stimulus subjects (SS) for different levels of characteristics (physical attractiveness, social skills, and social status) by the Brazilian and American sample. Panels **(A,B)** represent means for SS with low and high physical attractiveness, panels **(C,D)** represent means for SS with low and high social skills, and panels **(E,F)** represent means for SS with low and high social status. Panels **(A,C,E)** indicate the data of the Brazilian participants and panels **(B,D,F)** indicate the data of the American participants.

Participants ratings of SS accorded well with the high/low levels of three characteristics presented in the descriptions, providing a useful validity check. For each of the three stimulus characteristics (physical attractiveness, social skills, and current/prospective social status) the shift from low to high not only increased ratings of the three associated traits, but it also increased ratings of all traits (Figure 1). There are 27 contrasts in the top halves of Supplementary Tables 2–4; 24 are significant and show higher ratings for high level stimuli.

The analysis showed that SS with high levels of physical attractiveness were rated as having more attractive faces, bodies and being healthier than those with low levels of physical attractiveness [*F*_(1, 236)_ > 503.70; *p* < 0.001; *r* ≥ 0.83]. In addition, they were also rated as having good financial status, being more sociable, ambitious, and intelligent (0.59 > *r* ≥ 0.47), with weaker effects on their agreeableness (*r* = 0.26) and sincerity (*r* = 0.29). SS with high levels of social skills were rated as more sociable, agreeable and sincere than those with low levels of social skills [*F*_(1, 236)_ > 684.79; *p* < 0.001; *r* ≥ 0.86]. Moreover, they were also rated as being ambitious, intelligent, and healthy, and having attractive faces and bodies (0.69 > *r* ≥ 0.54), while the effect on financial status was not significant. SS with high levels of social status were perceived as having better financial status and were rated as more ambitious/hard working and intelligent than those with low levels of social status [*F*_(1, 236)_ > 393.07; *p* < 0.001; *r* ≥ 0.79]. In addition, they were rated as being more sociable (*r* = 0.61); effects on their agreeableness, facial attractiveness and health were weaker (0.33 > *r* ≥ 0.18), while effects on their body attractiveness and sincerity were not significant.

**Figure 2:**
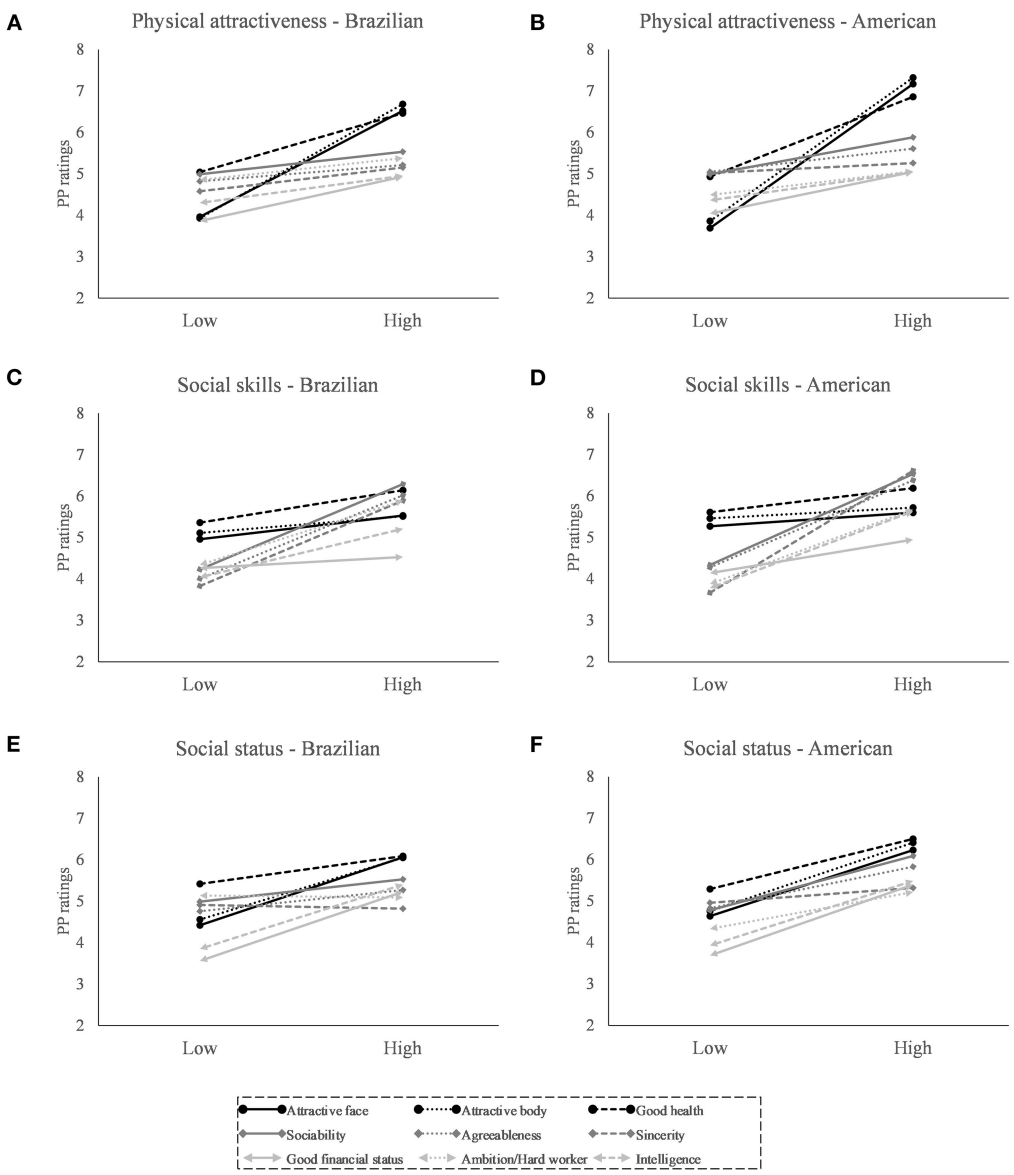
Means of traits of probable partners (PP) described for the stimuli subjects (SS) with different levels of characteristics (physical attractiveness, social skills, and social status) by the Brazilian and American sample. Panel **(A,B)** represent means for PP described for SS with low and high physical attractiveness, panel **(C,D)** represent means for PP described to SS with low and high social skills, and panel **(E,F)** represent means for PP described to SS with low and high social status. Panels **(A,C,E)** indicate the data of the Brazilian participants and panels **(B,D,F)** indicate the data of the American participants.

In general, participants ratings of PP suggested that they expected PP and SS to be similar on the three characteristics (physical attractiveness, social skills, and current/prospective social status) presented in the descriptions but, as for the SS, extended to other traits as well (Figure 2). There are 27 contrasts in the bottom halves of Supplementary Tables 2–4; 26 are significant and show higher ratings for the partners of stimuli with high-level characteristics.

For SS with high levels of physical attractiveness, participants rated the likely PP as having more attractive faces, bodies, and better health than for SS with low levels of physical attractiveness [*F*_(1, 236)_ > 424.86; *p* <0.001; *r* ≥ 0.80]. Their PP were also rated as having good financial status, and being more sociable, ambitious, and intelligent (0.72 > *r* ≥ 0.40), with weaker effects on their agreeableness (*r* = 0.39) and sincerity (*r* = 0.32). For SS with high levels of social skills, participants rated the likely PP as being more sociable, agreeable, and sincere than for SS with low levels of social skills [*F*_(1, 236)_ > 309.34; *p* < 0.001; *r* ≥ 0.75]. Their PP were also rated as being healthier, ambitious, and intelligent (0.77 > *r* ≥ 0.53), with weaker effects on financial status and physical attractiveness (0.39 > *r* ≥ 0.26). For SS with high levels of social status, participants rated the likely PP as having better financial status [*F*_(1, 236)_ = 213.15; *p* <0.001; *r* = 0.69], being more ambitious/hard working [*F*_(1, 236)_ = 20.60; *p* < 0.001; *r* = 0.28] and being more intelligent [*F*_(1, 236)_ = 314.49; *p* < 0.001; *r* = 0.76]. Their PP was also rated as being more sociable, agreeable, healthy, and having more attractive faces and bodies (0.80 > *r* ≥ 0.52); the effect on sincerity was not significant.

In summary, a high level in a given characteristic in the stimuli positively affected different traits in the SS and PP; similarity in couples was also observed for these traits. In addition, it was observed that male stimuli with high status are expected to be paired with PP with higher levels of physical attractiveness.

### Brazilian and American Samples Considered Separately

The interaction effects suggest that the subject descriptions were evaluated slightly differently in the two samples. For both the samples, SS with high levels of physical attractiveness were rated higher than SS with low levels of physical attractiveness for attractive face and sincerity; interaction effects indicated that the difference for attractive face was more pronounced in the American sample [*F*_(1, 236)_ = 25.30; *p* < 0.001; *r* = 0.31], while the difference for sincerity was stronger in the Brazilian sample [*F*_(1,236)_ = 9.39; *p* = 0.002; *r* = 0.20]. For PP paired to SS with high physical attractiveness, the participants provided higher ratings for the traits attractive face, attractive body and good health; interaction effects showed that most differences were more pronounced for the American sample [*F*_(1236)_ > 9.56; *p* < 0.002;0.20 ≤ *r* ≤ 0.28) (Figures 1, 2).

In general, SS with high levels of social skills were rated higher than SS having low levels of social skills for ambitious/hard working, intelligence and sincerity; interaction effects showed that the differences for ambitious/hard working and intelligence were more pronounced in the Brazilian sample [*F*_(1,236)_ > 8.67; *p* < 0.004; 0.19 ≤ *r* ≤ 0.24), while the difference for sincerity was stronger in the American sample [*F*_(1, 236)_ = 33.73; *p* < 0.001; *r* = 0.35]. Curiously, SS with low levels of social skills were rated higher among Americans for good financial status [*F*_(1, 236)_ = 21.57; *p* < 0.001; *r* = 0.29]. The PP of SS with high levels of social skills were rated higher for sincerity, good financial status and intelligence in both samples; the interaction effects showed that these differences were more pronounced in the American sample (Figures 1, 2).

In both samples, SS with high levels of social status were rated higher than those with low levels of social status for ambitious/hard working; interaction effects showed that these differences were more pronounced in the American sample [*F*_(1, 236)_ = 68.04; *p* < 0.001; *r* = 0.47]. The interactions also indicated that: SS with high levels of social status were also considered healthier and were rated higher for attractive body by the American participants, while the opposite effect was observed among Brazilians [*F*_(1, 236)_ > 19.56; *p* < 0.001; 0.28 ≤ *r* ≤ 0.39]; and that SS with high levels of social status were rated higher for attractive face by the Brazilians [*F*_(1, 236)_ = 12.56; *p* < 0.001; *r* = 0.22]. In contrast to the PP assigned to SS with low levels of social status, the PP for SS with high levels of social status were rated higher for good health, sociability, and agreeableness; interaction effects indicated that the differences for these traits were more pronounced for the American sample [*F*_(1, 236)_ > 9.60; *p* < 0.002; 0.20 ≤ *r* ≤ 0.29]. The Americans assigned more value to ambitious/hard working partners for SS with high levels social status [*F*_(1, 236)_ = 26.80; *p* < 0.001; *r* = 0.32]. See Supplementary Tables 2–4 for further details (Figures 1, 2).

In summary, most interactions showed small effects, indicating that male expectations of Brazilians and Americans are similar. That being said, Brazilians expected socially skilled men to have greater prospects for social status and American participants expected men with low social skills to have higher financial status. American participants expected men of high social status to be healthier and to have more attractive bodies; meanwhile, Brazilian males expected men of high social status to have more attractive faces and men of lower social status to have more attractive bodies. For both samples, male SS with the best characteristics were expected to have partners with better traits and these differences were more prominent for the American sample. As this effect on the expectations of the partners was observed for all characteristics (and only affected the intensity of rating), we believed that it may be a methodological artifact and not representing a specific trend for Americans or Brazilians.

## Discussion

This study examined whether male participants from two different countries exhibit similar mating expectations about heterosexual couple formation. Confirming our first prediction, the higher the level of a characteristic in the description of a stimulus male, the better the correspondent features were rated for their probable partner. These findings indicate that positive assortative mating is expected by our participants, similar to previous studies (Glicksohn and Golan, 2001; Jepsen and Jepsen, 2002; Silventoinen et al., 2003; Watson et al., 2004; Miller, 2007; Speakman et al., 2007; Castro et al., 2018; Conroy-Beam et al., 2019). The correspondence between the traits of the men and their partners suggests that high-quality individuals are expected to acquire high-quality partners, a prediction derived directly from the biological market perspective (Noë, 2017).

The predictions about the effect of a specific characteristic on the evaluation of different characteristics in men and their partners were also confirmed. Firstly, it was observed that high levels of physical attractiveness improved the ratings of male sociability and status traits; high levels of social skills enhanced the perception of male intelligence, ambition, and physical traits; and high social status increased male sociability. The effect of individual characteristics on other traits has also been well-documented in the literature (Anderson et al., 2001; Fink et al., 2006; Watkins, 2017; Castro et al., 2018; Wang et al., 2018). This finding suggests that individuals are assessed globally and that their mating value may be the result of the interactions of their characteristics, rather than the sum of the attributes. According to Conroy-Beam et al. (2019) in a context in which multiple preferences contribute to mate selection, assortative mating may have produced a specific pattern of desirability covariation in our species, thus a person who is desirable as a mate along any one preferred dimension tends to be desirable across all other dimensions. Secondly, the participant ratings showed that men of high social status were expected to be paired with partners having greater sociability, agreeableness, and physical quality (attractive face, body, and good health). In accord with the sexual strategies theory, men with high social status are expected to be paired with women who have high physical attractiveness, these traits correspond to the patterns of sexual preference typically found in our species (Buss and Schmitt, 1993; Dunkel et al., 2019).

The American and Brazilian mating expectations were similar; most of the differences found were non-significant or had small effects. Similarity for mating expectations is expected since the typical patterns of preference for mating are mostly similar across different cultures (Buss and Schmitt, 1993; Thomas et al., 2019; Walter et al., 2020). Considering that the samples have similar expectations, we propose to explore possible explanations for some of the small differences observed. Although this is not the purpose here, insights into these differences may be important for future work.

Brazilian participants considered men with high levels of social skills to be more ambitious and intelligent. The Brazilian self-image may contribute to the greater importance placed on good social skills. Culturally, Brazilian people tend to consider themselves to be highly affectionate, cordial, and hospitable (Carvalho, 2000; Miura et al., 2019), regardless of whether these beliefs are false or not, social ideals/expectations might have a direct effect on the evaluation of individual characteristics in the local mating marketplace, resulting in a better rating of traits associated with social interaction.

Other differences observed between American and Brazilian men indicate the influence of economic differences and cultural stereotypes on the local environment. American participants expected men of high status to be healthier, which may be related to the cost of access to health care (Schoen et al., 2010). Another factor that could contribute to this association is the financial barrier that low-income consumers face in maintaining a healthy diet in this part of America (Cassady et al., 2007). Among Brazilians, the link between financial status and access to health care might be less evident because of more access to public health care, which could influence the perceptions of safety concerning health issues among Brazilians—especially among younger adults (as in the current sample) that also tend to be in better physical condition.

The results also showed that Brazilians expect men of high status to have more attractive faces, while Americans expected these men to possess more attractive bodies; both are aspects of physical attractiveness that can be improved by those who have better financial condition. The positive relationship between physical attractiveness and status has already been observed specifically among men (Anderson et al., 2001). Curiously, Brazilian participants assigned more attractive bodies to men of lower status. This observation might have been expected because body attractiveness is especially valued among younger Brazilians that have not yet achieved financial independence, i.e., most of the college students sampled in this study (Iriart and Andrade, 2002). The practice of physical activity with the intention of increasing their attractiveness has already been observed among lower class adolescents in Brazil (Tavares et al., 2012). Finally, one stereotype was also identified among the American participants, that males with low levels of social skills were rated higher for good financial condition. This result could be evidence of the high-competence-low-warmth stereotype (Fiske et al., 2007).

The present findings are similar to those found in female samples: positive assortative mating was expected in couples, covariation in trait assessment was found, and the stereotype high-status-man paired with highly physically attractive woman was also observed. Several differences between the expectations of Brazilians and Americans were also consistent between the sexes (see Castro et al., 2018 for more details). The similarity between the genders in the different samples suggests that environmental (or even cultural) factors have a consistent effect on expectations.

One of the limitations of the current study is with respect to the participant samples that were used, undergraduates in their early 20's and mostly financially dependent on their parents. Broader sampling of participants from different backgrounds and age groups is needed to determine if there are other detectable trends and assess how generalized our current understanding is. Studies based on undergraduates could also lower the prospective cultural variation. The use of differently conceived SS might also reveal new information about mating expectations, while the use of photographs could improve the participants sensibility to the stimulus and accuracy or honesty of their evaluations. Personality and self-esteem evaluations might also help to describe the sample particularities expressed in mating expectations. In the present study, nine traits were used to reduce the fatigue effect. Investigations that address a greater number of traits, different traits, or similarity to demographic characteristics may bring new information about mating expectations. Another limitation was that the study investigated only expectations about the formation of heterosexual couples. Studies that investigate non-heterosexual couples can increase knowledge about the dynamics of human reproduction.

Expectations are beliefs about what should happen in the future and they develop from socialization, knowledge, and individual or collective experiences. Expectations modulate behavior, as they influence attention, interpretation, information retention affect people's attitudes and decisions (Olson et al., 1996). We can assume that mating expectations must develop from the beginning of sexual maturation as soon as sexual motives arise. Since different cultures can present unique responses when dealing with environmental adversities, different reproductive solutions can arise. Socialization and experience in these specific contexts could shape mating expectations and be maintained via cultural transmission.

In conclusion, despite socio-cultural differences, male expectations are in line with well-described sex-specific human mating preferences. Positive assortative mating was generally expected within couples; the quality of some characteristics affected the expectations of different traits in the individuals themselves and their partners; and, finally, different environmental conditions, and cultural ideals were responsible for small differences, that may be related to the local mating market. We would like to highlight that the present study investigated mating expectations (rather than preferences or choice). Mating expectations can be a rich approach to investigate socio-cultural aspects that are related to mating in different cultures once people tend to behave consistently to their expectations in many contexts.

## Data Availability Statement

The raw data supporting the conclusions of this article will be made available by the authors, without undue reservation.

## Ethics Statement

The studies involving human participants were reviewed and approved by Office of Research Human Subjects Committee Santa Barbara HUMAN SUBJECTS PROTOCOL SUBMISSION ID 12-560. The patients/participants provided their written informed consent to participate in this study.

## Author Contributions

FC, WH, MY, and FL contributed to the design of the research. FC, SG, and FL contributed to the implementation of the research. FC took the lead in writing the manuscript. All authors discussed the results and contributed to the final manuscript.

## Conflict of Interest

The authors declare that the research was conducted in the absence of any commercial or financial relationships that could be construed as a potential conflict of interest.
