# Supplementary material for: Male Mating Expectations in Brazilian and American Samples
Source: Front Psychol. 2021 Feb 10;12:617754. doi: 10.3389/fpsyg.2021.617754 (PMC7902785; doi:10.3389/fpsyg.2021.617754)
Supplement: Supplementary file 1 [file Table_1.docx]

**Supplementary data**

Table S1

*Stimulus Subject Descriptions*

| Stimulus subject: | Description: |
| --- | --- |
| SS1  Physical attractiveness: High  Social skills: High  Social status: High | M. P. is an easy-going, competent man. He is always helpful to other people even if he has to stop what he is doing. He became a government diplomat a few years ago. Now he has a good salary and few expenses so he has a lot of money to spend however he likes. Given his physical characteristics, many people find that M. P. is a very attractive man; some say it's hard to take their eyes off him. |
| SS2  Physical attractiveness: High  Social skills: High  Social status: Low | Several people believe that T. L. is extremely handsome. Many even use him as a reference when they want to describe a very good-looking man. He is a janitor; his job leaves him little free time and not much spending money. Everyone who knows him says that he is funny; people enjoy being around him. |
| SS3  Physical attractiveness: High  Social skills: Low  Social status: High | F. N. is a distinguished lawyer; his advice is valued by many large companies and he deals only with cases that involve a lot of money. Several of his acquaintances believe he could be a model. They also say that he is selfish, that he does not accept advice from others, and that he is always criticizing and finding flaws in other peoples’ work. |
| SS4  Physical attractiveness: High  Social skills: Low  Social status: Low | Women think R. P. is hot. He exercises regularly and takes good care of his health and his body. He is snob; he feels superior to others and is a know-it-all. He has few friends because he is very judgmental. He works as a telemarketer at a local company, working double shifts. Due to lack of time and a low salary, he cannot go out often with his friends. |
| SS5  Physical attractiveness: Low  Social skills: High  Social status: High | C. H. holds a high rank in the military where he coordinates several major operations. He earns much more than his same-age friends. He is ugly; people who know him say that looks are not his strong point. All his friends love him. They consider C.H. an amusing and easy-going person who is nevertheless very competent and responsible for his age. |
| SS6  Physical attractiveness: Low  Social skills: High  Social status: Low | D.M.’s appearance does not appeal to women. Many people say he looks strange. He is a nice, supportive man who is always smiling and willing to help his friends. He works as a salesperson in a shop near the city center. Financially speaking, his job is not very good because it pays poorly and does not allow him many luxuries. |
| SS7  Physical attractiveness: Low  Social skills: Low  Social status: High | In terms of physical attractiveness V. S. is well below average. Some people consider him asymmetrical and unpleasant to look at. Nobody likes to be around him because they consider him an irritating and shady person. He earns a high salary as a manager of a major industrial company. |
| SS8  Physical attractiveness: Low  Social skills: Low  Social status: Low | Women consider M. H. terrible looking. He is self-centered and unreliable. He does not have many friends and even his closest friends do not enjoy spending time with him. He works as a waiter in a coffee shop that is near his home. Although he enjoys his job, he does not earn much money. |

Table S2

*Mean Values and Contrast between the Ratings of Stimuli Subject with High and Low Physical Attractiveness*

|  |  |  | Brazilian |  |  | American |  |  | Profile | |  |  | Sample | |  |  | Profile*Sample | | | |
| --- | --- | --- | --- | --- | --- | --- | --- | --- | --- | --- | --- | --- | --- | --- | --- | --- | --- | --- | --- | --- |
|  |  |  | High *M*(*SD*) | Low *M*(*SD*) | | High *M*(*SD*) | Low *M*(*SD*) | | *F*(1, 236) | | *r* |  | *F*(1, 236) | | *r* |  | *F*(1, 236) | | *r* | |
| Stimulus-subject ratings | | |  |  |  |  |  |  |  | |  |  |  | |  |  |  | |  | |
|  | Physical attractiveness | |  |  |  |  |  |  |  | |  |  |  | |  |  |  | |  | |
|  |  | Attractive face | 6.93(1.35) | 2.52(1.22) |  | 7.77(0.99) | 2.21(0.86) |  | 1901.56 | *** | .94 |  | 7.69 | ** | .18 |  | 25.30 | *** | .31 |  |
|  |  | Attractive body | 7.19(1.28) | 2.31(1.13) |  | 7.70(0.96) | 2.92(1.00) |  | 1863.61 | *** | .94 |  | 37.00 | *** | .37 |  | 0.19 |  | .03 |  |
|  |  | Good health | 6.55(1.21) | 4.73(1.43) |  | 7.12(1.17) | 4.81(1.14) |  | 503.70 | *** | .83 |  | 5.72 | * | .15 |  | 6.94 | ** | .17 |  |
|  | Social skills | |  |  |  |  |  |  |  |  |  |  |  |  |  |  |  |  |  |  |
|  |  | Sociability | 5.19(1.05) | 4.58(1.01) |  | 5.27(0.85) | 4.54(0.76) |  | 93.36 | *** | .53 |  | 0.04 |  | .01 |  | 0.71 |  | .05 |  |
|  |  | Agreeableness | 4.70(1.03) | 4.38(1.13) |  | 4.77(0.86) | 4.58(0.72) |  | 17.16 | *** | .26 |  | 1.49 |  | .08 |  | 1.22 |  | .07 |  |
|  |  | Sincerity | 4.98(1.43) | 4.34(1.28) |  | 5.06(1.00) | 4.93(0.81) |  | 21.81 | *** | .29 |  | 6.43 | * | .16 |  | 9.39 | ** | .20 |  |
|  | Social status | | |  |  |  |  |  |  |  |  |  |  |  |  |  |  |  |  |  |
|  |  | Good financial status | 5.38(0.69) | 4.92(0.88) |  | 5.59(0.67) | 5.03(0.57) |  | 117.32 | *** | .58 |  | 3.95 | * | .13 |  | 1.35 |  | .08 |  |
|  |  | Ambitious/hard working | 6.44(1.21) | 6.07(1.36) |  | 6.41(1.04) | 5.69(0.95) |  | 65.61 | *** | .47 |  | 2.25 |  | .10 |  | 6.92 | ** | .17 |  |
|  |  | Intelligence | 5.45(0.90) | 4.85(1.25) |  | 5.83(0.87) | 5.30(0.97) |  | 79.95 | *** | .50 |  | 12.77 | *** | .23 |  | 0.26 |  | .03 |  |
| Probable partner ratings | | |  |  |  |  |  |  |  |  |  |  |  |  |  |  |  |  |  |  |
|  | Physical attractiveness | |  |  |  |  |  |  |  |  |  |  |  |  |  |  |  |  |  |  |
|  |  | Attractive face | 6.52(1.31) | 3.96(1.40) |  | 7.17(0.99) | 3.69(1.09) |  | 900.31 | *** | .89 |  | 2.41 |  | .10 |  | 20.80 | *** | .28 |  |
|  |  | Attractive body | 6.68(1.35) | 3.93(1.33) |  | 7.32(0.93) | 3.86(1.11) |  | 1028.38 | *** | .90 |  | 5.04 | * | .14 |  | 13.60 | *** | .23 |  |
|  |  | Good health | 6.46(1.30) | 5.04(1.36) |  | 6.86(1.15) | 4.93(1.16) |  | 424.86 | *** | .80 |  | 1.04 |  | .07 |  | 9.56 | ** | .20 |  |
|  | Social skills | |  |  |  |  |  |  |  |  |  |  |  |  |  |  |  |  |  |  |
|  |  | Sociability | 5.53(1.37) | 4.99(1.29) |  | 5.88(1.07) | 4.99(1.07) |  | 94.12 | *** | .53 |  | 1.57 |  | .08 |  | 5.59 | * | .15 |  |
|  |  | Agreeableness | 5.21(1.40) | 4.82(1.42) |  | 5.61(1.17) | 5.05(1.11) |  | 43.05 | *** | .39 |  | 4.21 | * | .13 |  | 1.33 |  | .07 |  |
|  |  | Sincerity | 5.15(1.40) | 4.58(1.39) |  | 5.26(1.14) | 5.02(1.12) |  | 27.71 | *** | .32 |  | 3.44 |  | .12 |  | 4.42 | * | .14 |  |
|  | Social status | | |  |  |  |  |  |  |  |  |  |  |  |  |  |  |  |  |  |
|  |  | Good financial status | 4.93(1.15) | 3.86(1.24) |  | 5.05(0.98) | 4.04(1.00) |  | 239.13 | *** | .71 |  | 1.32 |  | .07 |  | 0.13 |  | .02 |  |
|  |  | Ambitious/hard working | 5.38(1.33) | 4.85(1.35) |  | 5.06(1.17) | 4.49(1.15) |  | 45.14 | *** | .40 |  | 5.62 | * | .15 |  | 0.07 |  | .02 |  |
|  |  | Intelligence | 4.95(1.28) | 4.30(1.32) |  | 5.05(1.13) | 4.36(0.97) |  | 91.75 | *** | .53 |  | 0.36 |  | .04 |  | 0.10 |  | .02 |  |
| **p* < .05. ***p*< .01. ****p*< .001. | | | |  |  |  |  |  |  | |  |  |  | |  |  |  | |  | |

Table S3

*Mean Values and Contrast between the Ratings of Stimuli Subject with High and Low Social Skills*

|  |  |  | Brazilian |  |  | American |  |  | Profile | |  |  | Sample | |  |  | Profile*Sample | | |
| --- | --- | --- | --- | --- | --- | --- | --- | --- | --- | --- | --- | --- | --- | --- | --- | --- | --- | --- | --- |
|  |  |  | High *M*(*SD*) | Low *M*(*SD*) | | High *M*(*SD*) | Low *M*(*SD*) | | *F*(1, 236) | | *r* |  | *F*(1, 236) | | *r* |  | *F*(1, 236) | | *r* |
| Stimulus-subject ratings | | |  |  |  |  |  |  |  | |  |  |  | |  |  |  | |  |
|  | Social skills | |  |  |  |  |  |  |  | |  |  |  | |  |  |  | |  |
|  |  | Sociability | 7.18(1.34) | 2.58(1.29) |  | 7.14(1.11) | 2.67(0.94) |  | 1446.10 | *** | .93 |  | 0.04 |  | .01 |  | 0.26 |  | .03 |
|  |  | Agreeableness | 6.68(1.46) | 2.40(1.26) |  | 6.97(1.17) | 2.38(1.04) |  | 1313.11 | *** | .92 |  | 1.49 |  | .08 |  | 1.69 |  | .08 |
|  |  | Sincerity | 6.11(1.62) | 3.22(1.66) |  | 7.26(1.14) | 2.73(1.32) |  | 684.79 | *** | .86 |  | 6.43 | * | .16 |  | 33.73 | *** | .35 |
|  | Physical attractiveness | |  |  |  |  |  |  |  |  |  |  |  |  |  |  |  |  |  |
|  |  | Attractive face | 5.12(1.07) | 4.33(0.96) |  | 5.29(0.48) | 4.68(0.69) |  | 144.33 | *** | .62 |  | 7.69 | ** | .18 |  | 2.49 |  | .10 |
|  |  | Attractive body | 4.96(0.99) | 4.54(0.89) |  | 5.67(0.71) | 4.95(0.58) |  | 108.92 | *** | .56 |  | 37.00 | *** | .37 |  | 7.25 | ** | .17 |
|  |  | Good health | 5.98(1.36) | 5.30(1.13) |  | 6.31(0.99) | 5.61(1.09) |  | 96.48 | *** | .54 |  | 5.72 | * | .15 |  | 0.03 |  | .01 |
|  | Social status | | |  |  |  |  |  |  |  |  |  |  |  |  |  |  |  |  |
|  |  | Good financial status | 5.20(0.78) | 5.09(0.84) |  | 5.14(0.67) | 5.48(0.55) |  | 5.30 | * | .15 |  | 3.95 | * | .13 |  | 21.57 | *** | .29 |
|  |  | Ambitious/hard working | 6.95(1.29) | 5.56(1.37) |  | 6.46(1.10) | 5.65(0.91) |  | 207.81 | *** | .68 |  | 2.25 |  | .10 |  | 14.16 | *** | .24 |
|  |  | Intelligence | 5.59(1.10) | 4.71(1.11) |  | 5.82(0.98) | 5.32(0.85) |  | 113.12 | *** | .57 |  | 12.77 | *** | .23 |  | 8.67 | ** | .19 |
| Probable partner ratings | | |  |  |  |  |  |  |  |  |  |  |  |  |  |  |  |  |  |
|  | Social skills | |  |  |  |  |  |  |  |  |  |  |  |  |  |  |  |  |  |
|  |  | Sociability | 6.29(1.41) | 4.23(1.62) |  | 6.53(1.13) | 4.34(1.24) |  | 366.17 | *** | .78 |  | 1.57 |  | .08 |  | 0.32 |  | .04 |
|  |  | Agreeableness | 6.02(1.52) | 4.01(1.63) |  | 6.38(1.20) | 4.28(1.46) |  | 309.34 | *** | .75 |  | 4.21 | * | .13 |  | 0.13 |  | .02 |
|  |  | Sincerity | 5.89(1.54) | 3.83(1.52) |  | 6.61(1.35) | 3.67(1.33) |  | 457.75 | *** | .81 |  | 3.44 |  | .12 |  | 14.26 | *** | .24 |
|  | Physical attractiveness | |  |  |  |  |  |  |  |  |  |  |  |  |  |  |  |  |  |
|  |  | Attractive face | 5.53(1.16) | 4.96(1.39) |  | 5.60(0.85) | 5.27(0.94) |  | 32.67 | *** | .35 |  | 2.41 |  | .10 |  | 2.32 |  | .10 |
|  |  | Attractive body | 5.51(1.19) | 5.11(1.36) |  | 5.72(0.91) | 5.46(0.93) |  | 17.67 | *** | .26 |  | 5.04 | * | .14 |  | 0.78 |  | .06 |
|  |  | Good health | 6.14(1.33) | 5.36(1.30) |  | 6.19(1.08) | 5.61(1.10) |  | 93.57 | *** | .53 |  | 1.04 |  | .07 |  | 2.04 |  | .09 |
|  | Social status | | |  |  |  |  |  |  |  |  |  |  |  |  |  |  |  |  |
|  |  | Good financial status | 4.53(1.15) | 4.26(1.40) |  | 4.95(0.96) | 4.14(1.16) |  | 38.68 | *** | .38 |  | 1.32 |  | .07 |  | 9.55 | ** | .20 |
|  |  | Ambitious/hard working | 5.89(1.39) | 4.34(1.38) |  | 5.67(1.11) | 3.88(1.32) |  | 317.97 | *** | .76 |  | 5.62 | * | .15 |  | 1.80 |  | .09 |
|  |  | Intelligence | 5.21(1.34) | 4.04(1.41) |  | 5.63(1.06) | 3.78(1.18) |  | 291.25 | *** | .74 |  | 0.36 |  | .04 |  | 14.57 | *** | .24 |
| **p* < .05. ***p*< .01. ****p*< .001. | | | |  |  |  |  |  |  | |  |  |  | |  |  |  | |  |

Table S4

*Mean Values and Contrast between the Ratings of Stimuli Subject with High and Low Social Status*

|  |  |  | Brazilian |  |  | American |  |  | Profile | |  |  | Sample | |  |  | Profile*Sample | | |
| --- | --- | --- | --- | --- | --- | --- | --- | --- | --- | --- | --- | --- | --- | --- | --- | --- | --- | --- | --- |
|  |  |  | High *M*(*SD*) | Low *M*(*SD*) | | High *M*(*SD*) | Low *M*(*SD*) | | *F*(1, 236) | | *r* |  | *F*(1, 236) | | *r* |  | *F*(1, 236) | | *r* |
| Stimulus-subject ratings | | |  |  |  |  |  |  |  | |  |  |  | |  |  |  | |  |
|  | Social status | |  |  |  |  |  |  |  | |  |  |  | |  |  |  | |  |
|  |  | Good financial status | 7.66(1.09) | 2.63(0.99) |  | 7.84(0.79) | 2.78(0.87) |  | 2870.65 | *** | .96 |  | 3.95 | * | .13 |  | 0.02 |  | .01 |
|  |  | Ambitious/hard working | 6.83(1.41) | 5.68(1.42) |  | 7.45(0.96) | 4.66(1.26) |  | 393.07 | *** | .79 |  | 2.25 |  | .10 |  | 68.04 | *** | .47 |
|  |  | Intelligence | 6.77(1.22) | 3.53(1.25) |  | 7.24(0.97) | 3.90(1.11) |  | 1223.45 | *** | .92 |  | 12.77 | *** | .23 |  | 0.28 |  | .03 |
|  | Physical attractiveness | |  |  |  |  |  |  |  |  |  |  |  |  |  |  |  |  |  |
|  |  | Attractive face | 4.91(1.02) | 4.54(0.99) |  | 4.97(0.62) | 5.00(0.52) |  | 9.44 | ** | .20 |  | 7.69 | ** | .18 |  | 12.56 | *** | .22 |
|  |  | Attractive body | 4.55(1.02) | 4.95(0.84) |  | 5.49(0.85) | 5.14(0.49) |  | 0.19 |  | .03 |  | 37.00 | *** | .37 |  | 42.44 | *** | .39 |
|  |  | Good health | 5.59(1.38) | 5.69(1.05) |  | 6.20(1.00) | 5.73(1.07) |  | 8.21 | ** | .18 |  | 5.72 | * | .15 |  | 19.56 | *** | .28 |
|  | Social skills | | |  |  |  |  |  |  |  |  |  |  |  |  |  |  |  |  |
|  |  | Sociability | 5.19(1.07) | 4.58(0.94) |  | 5.33(0.72) | 4.47(0.80) |  | 138.00 | *** | .61 |  | 0.04 |  | .01 |  | 4.09 | * | .13 |
|  |  | Agreeableness | 4.62(1.16) | 4.47(1.01) |  | 4.92(0.80) | 4.43(0.76) |  | 26.42 | *** | .32 |  | 1.49 |  | .08 |  | 7.72 | ** | .18 |
|  |  | Sincerity | 4.62(1.36) | 4.70(1.24) |  | 5.15(0.93) | 4.83(0.90) |  | 2.63 |  | .10 |  | 6.43 | * | .16 |  | 7.62 | ** | .18 |
| Probable partner ratings | | |  |  |  |  |  |  |  |  |  |  |  |  |  |  |  |  |  |
|  | Social status | |  |  |  |  |  |  |  |  |  |  |  |  |  |  |  |  |  |
|  |  | Good financial status | 5.23(1.37) | 3.56(1.36) |  | 5.40(1.35) | 3.69(1.19) |  | 213.15 | *** | .69 |  | 1.32 |  | .07 |  | 0.04 |  | .01 |
|  |  | Ambitious/hard working | 5.09(1.45) | 5.14(1.23) |  | 5.22(1.32) | 4.33(1.18) |  | 20.60 | *** | .28 |  | 5.62 | * | .15 |  | 26.80 | *** | .32 |
|  |  | Intelligence | 5.40(1.48) | 3.85(1.21) |  | 5.49(1.09) | 3.93(1.19) |  | 314.49 | *** | .76 |  | 0.36 |  | .04 |  | <0.01 |  | <.01 |
|  | Physical attractiveness | |  |  |  |  |  |  |  |  |  |  |  |  |  |  |  |  |  |
|  |  | Attractive face | 6.06(1.42) | 4.42(1.21) |  | 6.23(0.92) | 4.64(0.81) |  | 380.18 | *** | .79 |  | 2.41 |  | .10 |  | 0.11 |  | .02 |
|  |  | Attractive body | 6.05(1.49) | 4.56(1.14) |  | 6.41(0.91) | 4.76(0.87) |  | 349.67 | *** | .77 |  | 5.04 | * | .14 |  | 0.86 |  | .06 |
|  |  | Good health | 6.09(1.40) | 5.42(1.23) |  | 6.50(0.93) | 5.29(1.22) |  | 176.95 | *** | .65 |  | 1.04 |  | .07 |  | 15.01 | *** | .24 |
|  | Social skills | | |  |  |  |  |  |  |  |  |  |  |  |  |  |  |  |  |
|  |  | Sociability | 5.53(1.43) | 4.99(1.28) |  | 6.09(0.97) | 4.78(1.21) |  | 131.40 | *** | .60 |  | 1.57 |  | .08 |  | 22.43 | *** | .29 |
|  |  | Agreeableness | 5.27(1.51) | 4.76(1.39) |  | 5.83(1.12) | 4.83(1.14) |  | 89.69 | *** | .52 |  | 4.21 | * | .13 |  | 9.60 | ** | .20 |
|  |  | Sincerity | 4.82(1.47) | 4.91(1.28) |  | 5.32(1.19) | 4.96(1.27) |  | 2.56 |  | .10 |  | 3.44 |  | .12 |  | 7.24 | ** | .17 |
| **p* < .05. ***p*< .01. ****p*< .001. | | | |  |  |  |  |  |  | |  |  |  | |  |  |  | |  |
